# Low Temperature Solution-Processable 3D-Patterned Charge Recombination Layer for Organic Tandem Solar Cells

**DOI:** 10.3390/ma12010162

**Published:** 2019-01-07

**Authors:** Jin Woo Choi, Jong Woo Jin, Denis Tondelier, Yvan Bonnassieux, Bernard Geffroy

**Affiliations:** 1 LPICM, CNRS, Ecole Polytechnique, Université Paris Saclay, 91128 Palaiseau, France; jinwoo.choi@polytechnique.edu (J.W.C.); jongwoo.jin@polytechnique.edu (J.W.J.); denis.tondelier@polytechnique.edu (D.T.); yvan.bonnassieux@polytechnique.edu (Y.B.); 2 LICSEN, NIMBE, CEA, CNRS, Université Paris-Saclay, CEA Saclay, 91191 Gif-sur-Yvette Cedex, France

**Keywords:** organic tandem solar cell, 3D nano-ripple pattern, ZnO sol-gel, charge recombination layer, low temperature solution process

## Abstract

We propose a novel method to pattern the charge recombination layer (CRL) with a low-temperature solution-processable ZnO layer (under 150 °C) for organic solar cell applications. Due to the optimal drying process and thermal annealing condition, ZnO sol-gel particles formed a three-Dimensional (3D) structure without using a high temperature or ramping method. The generated 3D nano-ripple pattern showed a height of around 120 nm, and a valley-to-valley distance of about 500 nm. Based on this newly developed ZnO nano-ripple patterning technique, it was possible to pattern the CRL without damaging the underneath layers in tandem structure. The use of nano-ripple patterned ZnO as the part of CRL, led to the concomitant improvement of the power conversion efficiency (PCE) of about 30%, compared with non-patterned CRL device.

## 1. Introduction

Finding alternatives for the current energy sources (i.e., burning fossil fuels, nuclear materials) has become one of the most important societal challenges for relieving the environmental pollution problem [1]. One of the most promising next-generation energy sources is solar energy, which can be converted to electric power via photovoltaic technology. Currently industrialized photovoltaic panels are based on inorganic materials such as silicon [2,3,4]. Recently, organic solar cells (OSCs) emerged as an alternative to inorganic photovoltaics devices [5,6,7]. The merits of OSC technology are: a low-cost solution process, a low temperature process, flexibility, and a tailorable material for further improvement. Recently, the champion single-junction OSC has reached a power conversion efficiency (PCE) of 12.6% [8]. However, it is still necessary for improving PCE and air-stability for large-scale commercialization.

One of the reasons for the low PCE is the narrow light absorption range of organic materials. A tandem solar cell structure, where two or more single-junction cells with complementary absorption spectra are connected in tandem, can be a promising design to overcome the limitations of single cells [9,10,11]. A tandem structure offers several advantages: (1) a broad absorption spectrum due to the usage of complementary absorbing materials; (2) summation of the open circuit voltage (V_OC_) of each sub-cell; (3) a reasonable fill factor (FF) due to higher optical density over a wider fraction of solar spectrum than that of single cell without increasing internal resistance. To maximize these advantages, the tandem device requires the qualified charge recombination layer (CRL) to simultaneously act as the anode for one of two adjacent sub-cells, and as the cathode for the other [12]. The CRL should have low electrical resistance, high optical transparency in the visible range, and a low barrier for charge recombination. Furthermore, the layer should be able to protect the lower layers during the remaining solution fabrication process.

In 2007, the CRL made with a metal oxide material was suggested [13]. The advantage of using a metal oxide as a part of CRL is to minimize the absorption at visible wavelengths. The classical structure of CRL is based on 0.5 nm LiF/1 nm Al/3 nm WoO_3_, used as multilayers. The first all-solution-processable tandem OSC was reported by Kim et al., where poly(3,4-ethylenedioxythiophene) polystyrene sulfonate (PEDOT:PSS) and TiO_X_ were used to form CRL [14]. In 2013, the first inverted tandem OSC that broke 10% efficiency was reported by You et al., with PEDOT:PSS and ZnO being used as CRL [15]. As shown above, new efficient CRLs with a large work function difference between two opposite interfaces have been developed continuously. Therefore, is high V_OC_ of the tandem device without loss-in-sum of the V_OC_ of the component cells is achieved. However, maximizing the short circuit current of tandem device is still under development, due to the difficulties in the current matching between the component cells. The current of tandem device is limited by the cell that produces the lower current. Therefore, it is important to increase the short circuit current of each component cell by enhancing the charge extraction properties of CRL. However, to our knowledge, there has been no reported research on the tandem device with patterned CRL, even though the 3D pattern could enhance the charge extraction capability of the CRL.

In the single junction solar cell, patterning of the charge-collecting layer (CCL) has been developed widely to maximize the charge extraction ratio [16,17,18,19,20,21]. However, previously proposed fabrication techniques for growing nano-wires, nano-rods, and nanoporous layers are not compatible with tandem structure fabrication. High thermal annealing conditions or vacuum processes should be avoided for all solution-processable tandem devices, to prevent the degradation of the films underneath. Meanwhile, a solution-processable ZnO nano-ripple pattern was firstly shown in a single sub-device by Yang et al. in 2009, by using a ramping thermal annealing method [22]. By using patterned ZnO CCL, the PCE improved by about 25% compared with device-containing planar CCL. However, the high thermal annealing treatment condition of this technique (275–350 °C) limited the use of this ripple patterning only in single inverted OSCs [22,23,24].

Herein, we found the optimal process conditions for nano-ripple patterning of the ZnO film in low-temperature conditions. After confirming the characteristics of ZnO in the single sub-cell device, the layer was introduced as a part of CRL in the tandem device. A newly developed low-temperature process technique actualized the patterning on the CRL without damaging the films underneath. As compared to the non-patterned device, the tandem device with ZnO ripple-patterned CRL showed a PCE improvement of about 30%.

## 2. Experimental Section

**Figure 1:**
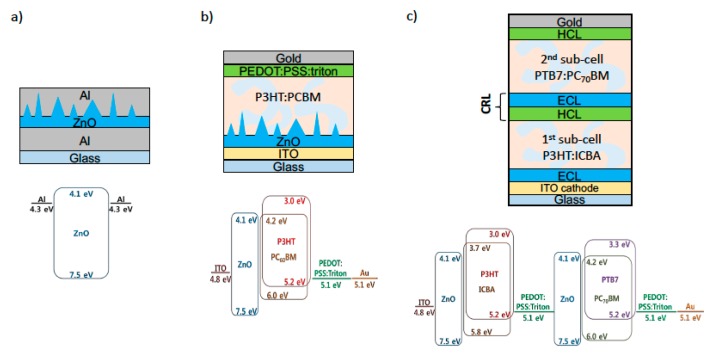
Device structures used in this study, with relevant energy levels of the different layers. (**a**) Electron only device, (**b**) single solar cell with a nano-ripple pattern, (**c**) tandem solar cell. ECL (electron collecting layer) and HCL (hole-collecting layer) are the ZnO nano-ripple layer and the PEDOT:PSS:Triton layer respectively.

As shown in Figure 1, for electron-only devices, ZnO films with different pattern sizes, single-junction OSCs, and tandem OSCs with a ZnO layer were fabricated and analyzed. The detailed fabrication methods are described below. 

### 2.1. Preparation of Solutions

A ZnO_X_ sol-gel solution was prepared by the following method. Zinc acetate dehydrate (ZnO precursor) at 1.5 M concentration was prepared in 2-methoxyethanol, followed by adding 0.1 mL of ethanolamine. Two types of PEDOT:PSS (Al4083 and PH1000, respectively) with surfactant were used as the hole-collecting layer. A volume of 100 µL of surfactant Triton was mixed with 4 mL of 1:1 volume ratio of AI4083 and PH1000. The ratio of PEDOT:PSS was 1:6 for Al4083, and 1:2.5 for PH1000. PH1000 contains higher amount of PEDOT, and thus has a higher conductivity than AI4083. AI4083 and PH1000 were purchased from Bayer (Leverkusen, Germany). In solar cells, poly(3-hexylthiophene) (P3HT) and poly[[4,8-bis[(2-ethylhexyl)oxy]benzo[1,2-b:4,5-b’]dithiophene-2,6-diyl][3-fluoro-2-[(2-ethylhexyl)carbonyl]thieno[3,4-b]thiophenediyl]] (PTB7) were used as the donor materials, while 1′,1″,4′,4″-tetrahydro-di[1,4]methanonaphthaleno[1,2:2′,3′,56,60:2″,3″][5,6]fullerene-C60 (ICBA), 6,6-phenyl C61-butyric acid methyl ester (PCBM), and 6,6-phenyl C71-butyric acid methyl ester (PC_70_BM) were used as acceptor materials. P3HT, PTB7, and fullerene derivatives were purchased from Ossila (Sheffield, UK). For a single solar cell (Figure 1b), a blend of P3HT and PCBM (1:1 weight ratio) was used as the active layer by dissolving 40 mg of P3HT:PCBM in 1 mL 1,2-dichlorobenzene (DCB, Sigma Aldrich, St. Louis, MO, USA). For the tandem solar cell (Figure 1c), the first sub-cell (bottom cell, Figure 1c) was based on a blend of P3HT and ICBA, and the second sub-cell (top cell, Figure 1c) was a blend of PTB7 and PC_70_BM. The P3HT:ICBA blend solution was prepared by dissolving 40 mg of P3HT:ICBA (1:1 weight ratio) in 1 mL of DCB. For second sub-cell active layer, the solution was prepared by dissolving 1:1.5 weight ratio of PTB7:PC_70_BM in DCB with a total concentration of 25 mg/mL. All prepared solutions were stirred for 12 h in a N_2_ glove box.

### 2.2. Electron-Only Device

An aluminum layer of 100 nm thickness was deposited onto glass substrates by thermal evaporation. The deposition rate of Al was 0.25 Å/s under 5 × 10^−7^ Torr process pressure. Prepared ZnO sol-gel solution was spin-coated on the Al deposited glass through a 0.45 μm pore polyvinylidene fluoride (PVDF) filter at 1000 rpm for 60 s. The obtained films were dried in ambient air over 5, 10, 30, 60, and 180 min, respectively, and then thermally annealed over 10 min at 125, 150, 175, and 200 °C, respectively. The final thickness of the films was 400 nm. For top electrode, 100 nm thick aluminum layer was thermally evaporated through a shadow mask, defining a device area of 0.28 cm^2^. The deposition condition of Al was same as the bottom electrode. ZnO film obtained by ramping method, as described by Yang group, was also prepared for comparison [21]. The current-voltage (I-V) characteristics of the ZnO electron-only device were measured by a Keithley 4200 (Tektronix, Beaverton, OR, USA). Atomic force microscopy (AFM) was used for the imaging of the 3D pattern of the fabricated samples.

### 2.3. Organic Solar Cell Fabrication and Characterization

OSCs were fabricated on ITO substrates (sheet resistance 20 Ω/square) purchased from Xinyan Technologies (Kowloon, Hong Kong). Pre-patterned ITO coated glass substrates were cleaned in an ultrasonic cleaner with deionized water and isopropyl alcohol (IPA) for 20 min each.

For single solar cell (Figure 1b), prepared ZnO sol-gel solution was deposited onto the ITO layer by spin coating (3000 rpm for 60 s) through a 0.45 μm pore PVDF filter in a N_2_ glove box. The substrates were directly transferred outside the glove box and dried in ambient air during different times (0, 5, 10, 30, 60, and 180 min, respectively) and then thermally annealed at 125 °C over 10 min, in order to form nano-ripple structures. The final thickness of the fabricated film was 200 nm. The samples were then transferred into N_2_ glove box to deposit the following layers. A P3HT:PCBM solution was filtered through a 0.45 μm pore polytetrafluoroethylene (PTFE) filter, and spin-coated in two steps (500 rpm for 30 s, and then 1000 rpm for 45 s). The films were thermally annealed at 145 °C for 10 min. The thickness of this layer was approximately 100 nm after annealing. Then, PEDOT:PSS:Triton solution was deposited at 1000 rpm for 60 s. The thickness of this layer was 120 nm after annealing at 120 °C for 2 min. 

For tandem cells (Figure 1c), the ZnO nano-ripple layer (ECL), the first sub-cell active layer, and PEDOT:PSS:Triton layer (HCL) were fabricated in the same way as reported above. PTB7:PC_70_BM was used as active layer in the second sub-cell. The PTB7:PC_70_BM solution was filtered through a 0.45 μm pore PTFE filter, and spin-coated at 700 rpm for 60 s. The layer was annealed in DCB solvent vapor for 5 min. The thickness of this layer was approximately 80 nm. Finally, a gold electrode (anode) was thermally evaporated through a shadow mask, defining a 0.28 cm^2^ active surface area. The deposition rate of Au was 0.5 Å/s under 5 × 10^−6^ Torr process pressure. 

The current density-voltage (J-V) characteristics of the OSCs were measured in a N_2_ glove box using a source meter (Keithley 2635, Tektronix, Beaverton, OR, USA) in the dark and under illumination. An Air-Mass 1.5 (AM 1.5) solar simulator with 100 mW cm^−2^ was used as the light source.

## 3. Results and Discussion

### 3.1. ZnO Electron Collection Layer

**Figure 2:**
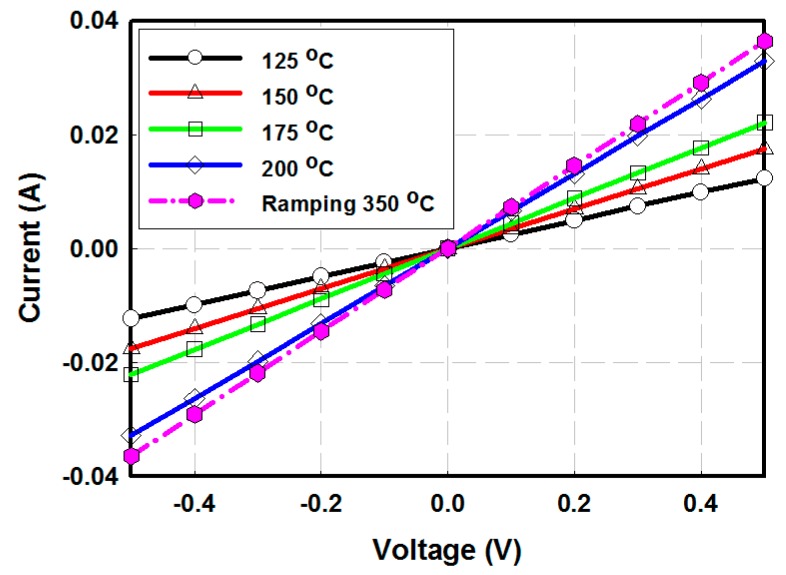
I-V characteristics of an Al (100 nm)/ZnO (400 nm)/Al (100 nm) electron-only diode device with a ZnO thickness of 400 nm.

To find the optimal annealing temperature for ZnO films, electron-only devices were fabricated to evaluate the conductivity of ZnO films under different thermal annealing conditions [25]. Figure 2 shows the I-V characteristics of Al/ZnO/Al electron-only device, with 400 nm thick ZnO. Table 1 summarizes the conductivity extracted from the I-V characteristics. The film made with the ramping fabrication method shows the highest conductivity among all films, but the difference is weak, and the conductivity values are in the same order of magnitude. A factor of 3 was observed between the lowest temperature annealing temperature (125 °C) and the ramping method (350 °C). It is obvious that the conductance of the ZnO thin film becomes higher when the film was annealed at higher temperature. Generally, a high annealing temperature provides a better crystallinity of the films, which directly impacts on the conductivity [26,27]. The reaction phenomena of ZnO sol-gel is reported in a previous report [28]. The ZnO precursor (zinc acetate dehydrate) film is highly resistive, because of the acetate functional group. Therefore, an annealing process is required to induce the reaction between the ZnO precursor with 2-methoxyethanol and the oxidation of a ZnO precursor in air. However, thermal annealing treatments of higher than 200 °C were not considered in this study, since the high annealing temperature of CRL could cause damage on the underlying layers in a tandem structure. It is then shown that the annealing temperature could be lowered to 125 °C.

### 3.2. ZnO Ripple Pattern

Based on the idea that ZnO ripple pattern growth mechanism is analogous to the coffee ring effect [27,29,30] and the solvent annealing technique [31], ZnO films were dried in the air at ambient temperature over various time periods (from 0 to 3 h) before annealing. After that, they were placed on a hot plate for thermal treatment over 10 min at 125, 150, 175, and 200 °C, respectively. Peak-to-valley roughness values (R_PtoV_) of patterned ZnO films, measured by AFM, are summarized in Table 2. The highest R_PtoV_ values for each annealing temperature were as follows: 110 nm at 125 °C, 122 nm at 150 °C, and 113 nm at 175 °C, achieved when the drying time was 60, 10, and 5 min, respectively. Devices with a short drying time require a high annealing temperature for the optimum pattern, i.e., the highest R_PtoV_ value. This result is reasonable, because short dried samples has more remaining solvent [32]. The sample will need a higher temperature to remove the remaining solvent, and consequently, to form the pattern. During the drying process, the films will partially dry, and small ZnO particles (cross-linked ZnO precursors) begin to form inside the film. These particles will travel, following the convection current of the solvent during thermal annealing of the film. Changing the drying time affects the initial size of seed crystal particles and the amount of the remaining solvent, which modifies the convection flow and the coffee ring effect [29,32]. The device with the highest R_PtoV_ was achieved at 150 °C after 10 min of drying time. The R_PtoV_ values were abruptly decreased with a long drying time (3 h) and a high annealing temperature (200 °C). The lack of remaining solvent due to the long drying process or fast evaporation at high temperature hinders the convection flow, and consequently, the rearrangement of ZnO precursor to form a 3D pattern. 

**Figure 3:**
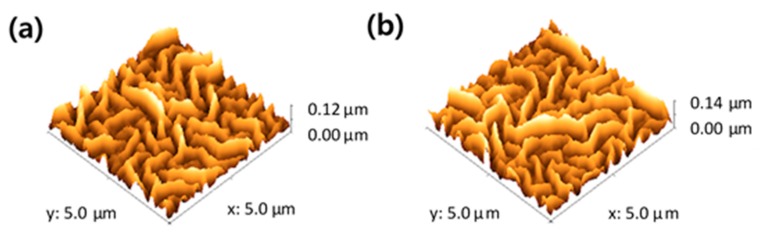
Atomic force microscopy (AFM) images of the ZnO nano-ripple, (**a**) the low temperature processed (10 min of drying time and annealing temperature of 150 °C) and (**b**) the ramping method.

The AFM images of the best nano-ripple pattern fabricated by a low temperature process, and the pattern obtained by ramping method, are shown in Figure 3. Pattern sizes with the novel low temperature method (Figure 3a) showed similar R_PtoV_ value to that achieved by the ramping method (Figure 3b). R_PtoV_ value of ZnO film fabricated by ramping method was 135 and 120 nm for drying time of 10 min followed by annealing temperature of 150 °C during 10 min. Finally, we succeed on the fabrication of 3D nano-patterned ZnO film, without using a high temperature, by introducing the drying step in the process. 

### 3.3. Single Junction Solar Cell with A 3D Nano-Patterned ZnO Layer

**Figure 4:**
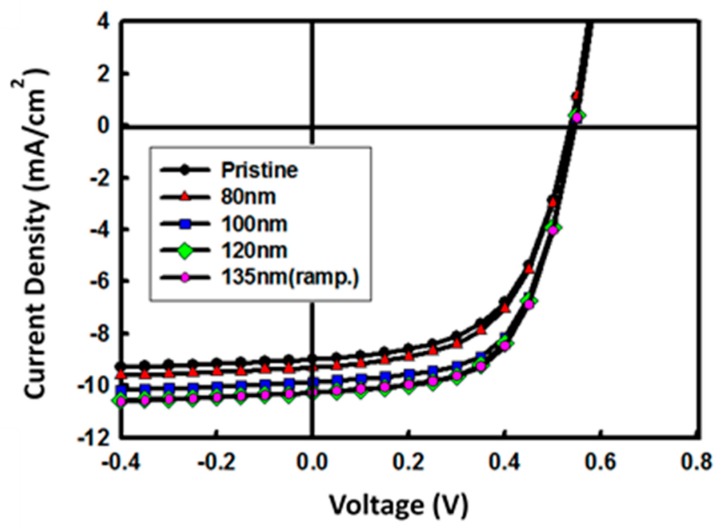
Current–voltage characteristics of the solar cells with different nano-patterned sizes of the ZnO electron-collecting layer (ECL). (ramp.) stands for the ramping method.

The inverted devices have a structure of ITO/ZnO/P3HT:PCBM (200 nm)/PEDOT:PSS (100 nm)/Al (100 nm) with a different ripple size of the ZnO layer. Figure 4 shows J-V characteristics of the inverted devices made with P3HT:PCBM under 100 mW/cm^2^ illumination. The performance characteristics of the OSCs are summarized in Table 3. Devices A to D were fabricated in a low-temperature process, with an annealing temperature of 150 °C for 10 min and different drying times. The drying time was 3000, 300, 60, and 10 min for devices A, B, C, and D, respectively. As previously shown in Table 2, the drying time modifies the pattern size (R_PtoV_) for a fixed temperature. Only drying time was controlled to change the 3D pattern size (R_PtoV_), in order to exclude the effects of the annealing temperature on the film properties, such as conductivity. The J-V curve that is labeled device E shows the characteristics of OSC, with the ZnO layer being made by the ramping method. Even though the ramping method could give a slightly better conductivity as shown in Table 1, the overall characteristic of device D shows similar characteristic with device E. The short circuit current (J_SC_) and FF increase as the ZnO pattern size increases, while V_OC_ does not show a significant change. As a result, the device with 120 nm R_PtoV_ has the best performance, with 3.4% of PCE. The improvements on the J_SC_ and FF are based on the efficient charge collection properties, by introducing a patterned ZnO layer. Due to the efficient carrier extraction, the space charge-inducing resistance near ECL will be reduced. When the pattern size of ZnO increases from 80 to 120 nm, an expectable result is a decrease in the series resistance. The series resistances extracted by the slope of the J-V curve at the V_OC_ were 27, 25, 21, and 21 Ω∙cm^2^ for device A, B, C, and D, respectively.

To assess the effect of ZnO layer on the electrical properties of the device more deeply, we examined the charge collection probability (P_collection_) in the cells, according to the method proposed by Kyaw et al. [33]. A photo-generated current (J_ph_) is saturated at the high internal voltage (V_int_) region, due to the large enough internal field extracted all generated charges. Therefore, the saturated photocurrent (J_ph,saturation_) can be written as following equation, while the value is limited only by the number of absorbed photons:

J_ph,saturation_ = qLG_max_ (1)

q is the elementary charge, L is the thickness of the active layer, and G_max_ is the maximum photoinduced carrier generation rate per unit volume. However, in a low internal voltage region, the J_ph_ is proportional to the P_collection_ in the cells. Therefore, the equation 1 can be written as:

J_ph,saturation_ = qLG_max_ P_collection_ (2)

From Equations (1) and (2), P_collection_ can be calculated by normalizing J_ph_ with J_ph,saturation_.

**Figure 5:**
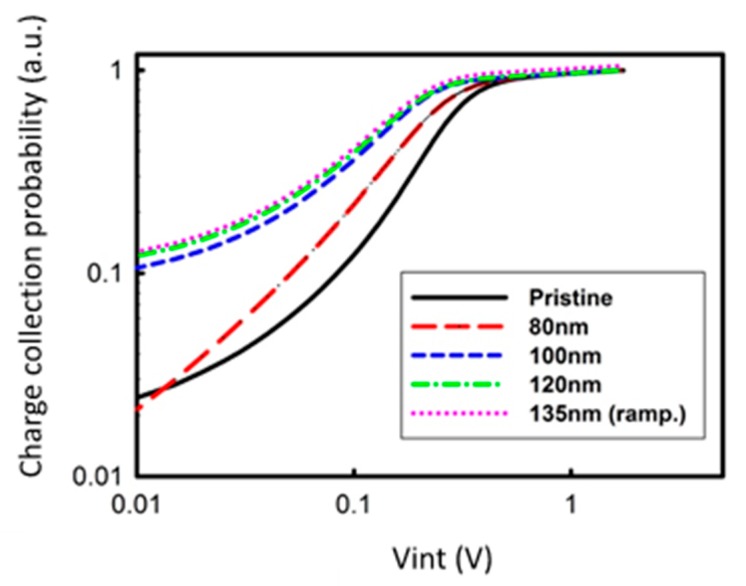
Charge collection probability as a function of the internal voltage for cells with different ZnO nano-pattern size.

Figure 5 shows the charge collection probability with respect to internal voltage (V_int_) under illumination of 100 mW/cm^2^. As shown in Figure 5, the overall charge collection probability of the inverted devices with ZnO increased, as the pattern size increases. The increment in charge collection probability is more significant at low V_int_ (high applied voltage). The observed increment of charge collection probability in the nano-patterned ZnO devices is well-matched with the J_SC_ characteristic of the OSCs. 

### 3.4. Tandem Solar Cell with Low-Temperature Processed ZnO

**Figure 6:**
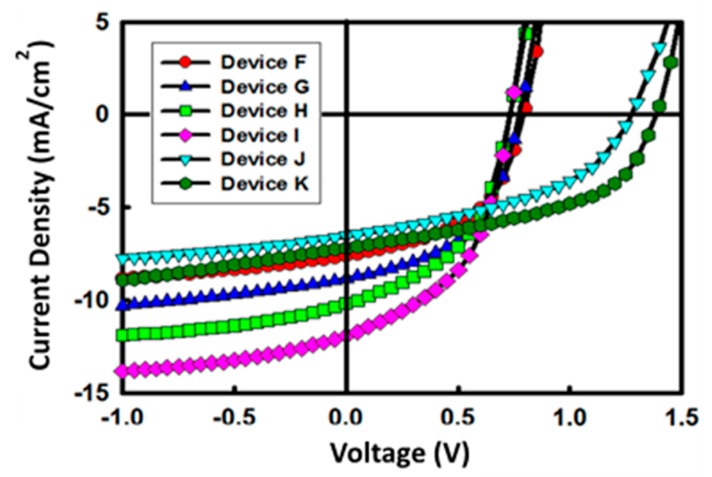
The J-V characteristics of the OSC devices with and without a ZnO patterned layer as ECL.

Photoactive layers with complementary absorption range were used as sub-cells of the tandem device. P3HT:ICBA was selected as the active layer for the bottom cell (device F and G). ICBA was selected as the acceptor, because the P3HT:ICBA active layer forms its optimal morphology in higher annealing temperature when compared with P3HT:PCBM [34,35,36]. Therefore, the P3HT:ICBA system promises a lower thermal degradation for tandem device fabrication, compared with the P3HT:PCBM system. The PTB7:PC_70_BM active layer is selected for the top cell (device H and I). Instead of thermal annealing, the solvent annealing method was used for the PTB7:PC_70_BM active layer, to minimize the thermal effect on the underneath layers in the tandem device process [37]. The sub-cells have the structure: ITO/ZnO (150 nm)/P3HT:ICBA (100 nm) or PTB7:PC_70_BM (150 nm)/PEDOT:PSS (120 nm)/Au (100 nm). The details of structure and photovoltaic devices performance are summarized in Table 4. Figure 6 presents the effect of the ZnO ripple pattern on the performance of each sub-cell. Among the devices F to I, devices with patterned ZnO show better J_SC_ and PCE. Likewise in the device with P3HT:PCBM, the increment of charge collection probability in the nano-patterned ZnO layer is the origin of these improvements. The inverted tandem devices have a structure of ITO/ZnO (150 nm)/P3HT:ICBA (100 nm)/PEDOT:PSS (120 nm)/ZnO (150 nm)/PTB7:PC_70_BM (150 nm)/PEDOT:PSS/Au (100 nm). The V_OC_ values of fabricated tandem devices are much higher than V_OC_ of each sub-cell (Table 4). This means that the sub-cells were series connected through CRL. V_OC_ of P3HT:ICBA device and PTB7:PC_70_BM device is 0.78 and 0.73 V, respectively. Therefore, ideally expected V_OC_ for tandem device is 1.5 V. Comparing this ideal value with V_OC_ of the fabricated tandem devices, there was only slight loss at around 0.1 to 0.2 V. A tandem device with a 3D nano-patterned ZnO (device K) shows a higher V_OC_, J_SC_, and FF than the device with pristine ZnO (device J). A better work function matching of CRL with active layers, efficient electron extraction, and electron-hole balance in the device are the reasons for this improvement [38,39]. A highly efficient carrier recombination characteristic of the CRL in a patterned ZnO layer reduced charge accumulation effect. Finally, the best device achieved a V_OC_ of 1.38 V, a J_SC_ of −7.2 mA/cm^2^, a FF of 49%, and PCE of 4.8%.

## 4. Conclusions

We developed a new method for patterning the ZnO layer by a low temperature (under 150 °C) solution process. A solution-processable ZnO layer shows proper electrical characteristics for OSCs even with a low temperature process. Nano-ripple pattern was fabricated to maximize the interface between the ZnO layer and the photoactive layer. In a single-junction device, PCE was about 30% higher when the ZnO layer was patterned. Finally, developed low-temperature processable nano-patterned ZnO was introduced in the tandem structure. The best tandem device has V_OC_ of 1.38 V and PCE of 4.8%, showing nearby 30% PCE improvement, compared to the non-patterned injection layer. It is shown that main improvement results from the charge extraction probability improvement, due to the nano-ripple patterned layer.

## Figures and Tables

**Table 1:** Conductivity value of the ZnO films under different annealing temperatures.

Temperature	125 °C	150 °C	175 °C	200 °C	Ramping 350 °C
Conductivity (S/m)	2.5 × 10^−3^	3.5 × 10^−3^	4.4 × 10^−3^	6.7 × 10^−3^	7.3 × 10^−3^

**Table 2:** R_PtoV_ value of ZnO films with different drying times and annealing temperatures (units in nm).

Drying Time	Annealing Temperature			
	125 °C	150 °C	175 °C	200 °C
0 min	78	109	93	21
5 min	84	111	113	22
10 min	96	122	110	19
30 min	102	106	99	21
60 min	110	98	93	22
3 h	43	87	83	21

**Table 3:** The summary of the key parameters of the single junction organic solar cells (OSCs), with different nano-ripple pattern sizes. Devices A to D were fabricated by the low-temperature method, while device E was fabricated by the ramping method.

Device X	Pattern Size (nm)	Voc (V)	J_SC_ (mA/cm^2^)	FF (%)	PCE (%)	Rs (Ω∙cm^2^)
**A**	0	0.54	−9.0	56	2.7	27
**B**	80	0.54	−9.3	56	2.8	25
**C**	100	0.55	−9.9	61	3.3	21
**D**	120	0.55	−10.3	60	3.4	21
**E**	135	0.55	−10.3	61	3.4	21

**Table 4:** The summary of the key parameters of the OSCs extracted from the J-V measurements. ECL stands for the electron collecting layer (ZnO layer).

Device	Structure	ECL (ZnO Layer)	V_OC_ (V)	Jsc (mA/cm^2^)	FF (%)	PCE (%)
**F**	First sub-cell	Non-patterned	0.79	−7.6	51	3.1
**G**		Nano-ripple	0.78	−8.8	50	3.5
**H**	Second sub-cell	Non-patterned	0.73	−10.2	48	3.6
**I**		Nano-ripple	0.73	−11.9	48	4.2
**J**	Tandem cell	Non-patterned	1.28	−6.5	44	3.7
**K**		Nano-ripple	1.38	−7.2	49	4.8
